# On the Insanity of Early Life

**Published:** 1857-10-01

**Authors:** A. Brierre de Boismont

**Affiliations:** Chevalier des Ordres de la Légion d'Honneur et du Mérite Militaire de Pologne; Ancien Médecin des Hôpitaux de Paris et de Varsovie; Lauréat de l'Institut et de l'Académie Impériale de Médecine, &c. &c.


### Art. II.—ON THE INSANITY OF EARLY LIFE.

BY M. BRIERRE DE BOISMONT, M.D.

Chevalier des Ordres de la Légion d'Honneur et du Mérite Militaire de Pologne;

Ancien Médecin des Hôpitaux de Paris et de Varsovie;

Lauréat de l'Institut et de l'Académie Impériale de Médecine, &c. &c.

[*Written expressly for this Journal.*]

THERE have recently been published in France two theses—one upon mental affections in children;* the other upon insanity at the epoch of puberty.† It seemed necessary to inquire the limit of the age of the individuals who formed the subjects of these two essays, because until then we had believed mental alienation to be very rare in childhood. Amongst the seventeen cases observed by the authors, the youngest were fourteen years of age, and the others varied from fifteen to twenty-two. The critic might reasonably object that the designation of children was scarcely applicable to the greater part of these patients. We are aware, however, that Haslam, Greding, Frank, Burrows, Spurzheim, Friedreich, Esquirol, and Guislain have related cases of insanity amongst children of less than eleven years. We owe to Dr. Marc, physician to Louis Philippe, the very curious observation of a young girl aged eight years, who openly avowed her intention to kill her mother, father, and

* Paulmier. Paris, 1856. † Rousseau. Paris, 1857.

grandmother. Two motives seemed to influence her in this resolution—the desire to *possess their property*, and to amuse herself with little boys and men. She was morose, taciturn, and answered very laconically to any questions addressed to her. In the country she abandoned herself early to solitary vice, without her health appearing to suffer; but on her return to the town, she began to fall away rapidly. It was some time before the cause of this emaciation was discovered: on surprit enfin ses habitudes onaniques; elle les confessa cyniquement, en disant qu'elle regrettait de ne pouvoir y substituer le commerce des petits garçons.*

In the course of a practice of more than thirty years, we have only observed three cases of mental derangement in children. The first relates to a pretty and intelligent little girl of seven years of age. Her mother was under treatment for a mental affection, and it was observed shortly that the child became irritable and capricious, and gave way to the most violent fits of passion, during which she would break and destroy everything which came to hand. Soon afterwards she became subject to attacks of ecstasy, in the course of which her features had a seraphic expression, and her eyes remained fixed upon the sky for a great length of time; she would cry aloud with a voice vibrating with emotion, "I see the angels; they are coming to me." When the crisis was past, she was very excitable for some time, but gradually became tranquil, and could answer rationally the questions put to her.

The second case was that of a boy, aged six, extremely difficult to manage, and of an irritability which had become, during the past four months, insupportable. When he was placed under my care, he could not remain in one place, was continually mounting upon the chairs, tables, and window-seats, and rolling in the dust; he ate gluttonously and irregularly. He would listen to nothing, but got into a rage if any one wished to control him. He perpetually escaped from surveillance, and was never found again until he had accomplished some mischief. On account of his violence, which rendered some serious result not improbable, it was necessary to impose mechanical restraint upon him. When he found himself thus disabled, he became enraged, and menaced us in a manner most extraordinary for a child of that age: "As soon as I am at liberty, I will set fire to the house, and if I can find a pointed knife, I will stab you to the heart; I should rejoice to see your blood flow, and to kill you." In his father's house, he had often used similar language; and it was the fear on the part of his parents that he would at

* *Marc.* De la Folie, t. i. p. 96.

some time carry his threats into execution, that had led them to the resolution of placing him in our institution. We found it would be dangerous to keep such a patient; he therefore returned home, and we lost sight of him.

The third example of this kind was observed by us in the asylum of St. Athanasius, founded by the much-regretted Dr. Follet. When we visited this model establishment, the directing physician, M. Baume, showed us a boy of ten years of age, who, notwithstanding a defect of the right eye, had a lively, bold, intelligent aspect; he was properly developed for that age. We were informed that he had an excellent memory, and learnt his lessons very easily. He had just made his first communion, and it was hoped that this religious act would have a favourable influence upon his shocking propensities. From his earliest years he had manifested the very worst instincts; he stole everything to which he took any fancy; he was the terror of his play-fellows, whom he pinched, struck, and abused in every way; he obeyed no orders, and wandered about incessantly. His parents had never been affected with mental disturbance, and he was an only child, so that jealousy could have no share in producing these results. His instincts became more and more perverted, and as he uttered threats perpetually, would strike and try to wound, and talked continually of killing some one, his mother determined to bring him to the asylum. There he became the terror and scourge of the patients, always pinching, biting, and striking. His victims were especially the imbecile and idiots. This kind of instinct exists also amongst these classes particularly. Last year, visiting an asylum, I saw, in the section devoted to idiots, one of them, who thought he was unobserved, steal round to give a kick to one of his companions, who had in no way molested him.

When the boy was in our presence, he seemed at first a little abashed, and spoke only in monosyllables. But speaking to him with much precaution, and attributing his misdeeds to his malady, he became more communicative, and answered our questions. He avowed quietly all that he had done; he said, "I have no pleasure except in doing mischief. I should like to shed your blood. When I pushed against my mother, it was to throw her down." On different occasions he manifested a desire to stab her with a knife to kill her. It is naturally, and without anger, that he does wrong. He knows well that it is wrong, but he feels no regret; he gives a blow as another child would give a piece of bread to a beggar. He spoke to us without reserve. One would have thought that the conversation was upon the most indifferent matters; the eyes had no particular expression. He retains the remembrance of what he supposes to be an injury, or of an unpremeditated wrong, and avenges it on the first opportunity. Religion has made the first attempt at cure ; a prolonged moral treatment may second it. It will be interesting to know what will result from this innate tendency to evil, against which chastisement would assuredly be inefficacious, independent of its injustice; and which would certainly, to any enlightened mind, be a surety of nonresponsibility in the commission of any criminal act.

These three cases establish clearly the fact that mental derangements may occur in childhood; but they constitute rather perversions of instinct, of sentiment, and of the moral faculties, than well-defined types of mania or monomania. This tendency, moreover, is in relation with the psychological dispositions of this period of life. For ninety-nine years there have been received at the Salpêtrière and Bicêtre, in the department of epileptics, idiots, and imbeciles, young children, who, examined carefully, do not really belong to this division, but are liars and thieves ; immodest and vicious in every form. M. Schnepf, in his thesis on Aberrations of Sentiment (1855), has related nine cases, among which are found children of seven years and nine years and a half.

Sundry authors, and amongst others, MM. Parchappe, Aubanel, Chore, Delâsiauve, and Paulmier, have classed mania amongst those affections to which children may be liable. The cases which we have seen, characterised by great agitation, have not appeared to us to constitute true mania ; and the communication made to the Medico-Psychological Society by Dr. Delasiauve refers chiefly to epileptic children, whose maniacal attacks were complicated by a kind of ecstasy. It is, however, necessary to recognise that a form of mania may exist in children. Lastly, in his Report of the Devon Asylum for 1856, Dr. Bucknill, after having divided insanity, according to the symptoms, into mania and melancholy, relates, in the first category, the case of a child, twelve years of age, who was brought to the asylum for having attempted suicide by drowning and strangulation. He was affected then with chorea. Around his neck was distinctly visible the mark produced by the cord. He cried incessantly, "I wish to die—I wish to die." He struck his head against the wall and tried to suffocate himself by pressing his fist against his throat. He bit and struck at every one who came near him. He was put in the padded room, and had baths and medicines to procure sleep. In forty-eight hours he was quieter. Three days after, the remedies having been discontinued, the symptoms returned with all their first violence, but yielded completely to hot baths, morphia, and cold affusion to the head.

The preceding observations leave no doubt as to the disorders of mind which may affect children. As yet the subject is new, and has not generally engaged attention; and it is easily to be understood how it happens that there is no large collection of such cases. But the subject being opened out, it is not to be doubted that shortly more extended and complete communications will furnish to education, to medicine, and to philosophy, new materials and useful data, which will rectify many errors. We may consult on this subject a very interesting essay by Dr. Bush.* The author divides the cases which he has observed into two series; 1st, those children who present excessive irritability of the nervous system, with a general lack of mental and bodily vigour; and 2nd, those who, with the same lack of vigour, present diminished irritability. After examining with the greatest care the causes of the physical, mental, and moral inequality of children, he shows forth the general standard of education to which all these varieties of intelligence are subjected. He shows then that before punishing idleness, inattention, obstinacy, perversity, but especially moral derelictions, as lying, theft, &c., we should most carefully examine whether these dispositions are due to education or to the defective nature of the child. Punishment, in this case, would be only an aggravation of the evil, whilst the best corrective would be modification or change of education. No doubt the custom of considering children as mere similar units of society makes a great proportion of them entirely ignorant, where it does not morally degrade them. But how few parents would be able to have private instructors? Their assemblage in communities is the most practicable resource; but it will never be advantageous to the country at large, until the heads of colleges and similar institutions devote themselves less to the making of money, and more to the careful consideration of the faculties of their pupils, with the view of leading each in his own peculiar vocation.

We arrive now at the second division of our subject, which treats particularly of mental alienation at the period of puberty, or rather of adolescence. Documents on this branch of inquiry are doubtless less rare, and the related facts much more numerous, but there does not exist as yet in France any good description of this phase of insanity. We are indebted nevertheless to Dr. Wigan, author of the "Duality of the Mind," for a remarkable notice on motiveless crime amongst young people.† These series of reprehensible acts belong very evidently to perversions of instincts and sentiments of which we have already spoken. Amongst the cases which the author has collected, we find

* On "Juvenile Delinquency." P. 57. Jour. 1849, P. 428.
† Journal of Psychological Medicine, &c., vol. ii. p. 497. Lond. 1849.

instances of incendiarism, poisoning, cruelty to animals and children, and even of murder. The age of these delinquents is generally sixteen to eighteen years amongst the girls, and from seventeen to twenty-one of the boys. The special characteristic of these acts is that of being uninfluenced by any motive of animosity towards the object injured.

According to the observation of Dr. Wigan, the majority of these young persons had been subject to nasal hæmorrhage, which in some cases, even in boys, appeared with the regularity of the menstrual secretion. The criminal act was generally committed after the temporary cessation of the flux. The aspect was then always heavy, stupid, and languid. In no case was there any animation of feature, nor any of the repulsive characters of vice.

On interrogation as to the motives of their conduct, they would answer almost invariably, "I do not know—I had no motive—I thought I must do it." No other answer could be obtained but this, "I was compelled to do something." As to the *something* itself, it was determined by a simple accident, the sight of the means of accomplishing it. Dr. Wigan attributes this irresistible impulse to a local and special congestion of the brain. He has observed facts of this nature in the most respectable families, in which the greatest pains had been taken, by education and example, to instil good principles into the minds of the children. To the same disposition of mind he attributes certain forms of forgetfulness of the rules of propriety and decency in society, contempt of public opinion, and rashness, which nothing could explain or justify. He relates also certain instances of bravery almost beyond precedent, which have excited to the utmost the admiration and applause of contemporaries, which were neither excited by rivalry, nor the love of glory, nor the desire of applause, nor the passion of war—but simply by this irresistible impulse to *do something*. Under this temporary constitutional impulse these young people have shown a contempt for dangers which at another time would have been incomprehensible, and which has excited in them veritable terror on reflection. Dr. Wigan's hypothesis is, that this state may result from the too slow growth of the osseous case of the brain. We do not discuss the hypothesis; we merely collate the facts, which have an important significance in relation to morals, education, and legal medicine. We may, however, remark, that the question in reference to causation is rather superficially treated; and that to be satisfactory, it would be necessary to obtain more precise information upon hereditary influences, upon the early maladies which might have modified the constitution, and especially upon the kind of education. We now pass on to our own observations.

The cases observed by us are 42 in number, which, out of 1200 patients received during the same time, give a proportion of about 1 in 28. This proportion may doubtless vary with greater numbers for comparison, but there is nothing surprising in it, inasmuch as it is strictly conformable to the calculation of probabilities: of these 42 insane persons, 23 were men, 19 women; their age was from 14 to 56 years, and they are in detail as follows:—

| Years of Age. | No. of Patients. | Years of Age. | No. of Patients. |
|---|---|---|---|
| 14 | 1 | 23 | 0 |
| 15 | 3 | 24 | 1 |
| 16 | 3 | 25 | 2 |
| 17 | 6 | 26 | 4 |
| 18 | 4 | 30 | 2 |
| 19 | 3 | 36 | 1 |
| 20 | 4 | 40 | 1 |
| 21 | 1 | 45 | 1 |
| 22 | 4 | 56 | 1 |

We shall here make an observation which may appear superfluous—that is, that the age indicated is that of the *last entry*; and that of all these cases, there is not one whose mental derangement did not date from childhood, from puberty, or from menstruation.

The first symptoms of the appearance of mental disorder among the boys, have generally been observed about the twelfth, thirteenth, fourteenth, and fifteenth years; and those cases in which they have not manifested themselves until the seventeenth or eighteenth year, had been in their earlier age *bizarres*, singular, unequal, capricious, and the children of diseased parents. Amongst the girls, the morbid phenomena generally first appeared at the eleventh, fourteenth, fifteenth, and sixteenth years; and those in whom they did not appear until the seventeenth and eighteenth, were characterized by similar states to those just mentioned, or had difficult menstruation. If true mental alienation begins in some of them about thirteen, fourteen, or fifteen years of age, we may affirm that in the majority of cases, the phenomena first observed are of a nervous, hysterical or convulsive nature—an unequal, depressed, eccentric disposition—in short, rather the elements or precursors of insanity, than the malady itself, which generally has a period of incubation more or less prolonged.

It is natural to inquire whether the causes of adult insanity are the same as those which determine that of the young; or if in the latter cases there may be peculiarities of organization, or special circumstances which modify the malady—the answer must be sought in the antecedents.

With the greatest care in inquiring into the causes or predisposing influences, there are twelve of the cases in which we have been unable to gather any precise information : perhaps because the parents have not attended to the early indications—a very common occurrence. There is nothing more frequent, indeed, than to be told by them, that the affection is quite recent, when the simplest questions oblige them to recognise the existence of certain signs long existing, often because they wilfully ignore any acknowledgment of hereditary influence.

There remain, then, thirty cases in which we have been able to collect precise information. Hereditary (13) and moral (5) causes have been noticed eighteen times. Independent of mental alienation, the parents were often eccentric, *bizzares*, of an excessive weakness, false in judgment ; either incapable of forming any decision, or of extreme obstinacy. More than once, the tendency to insanity has been denied ; but it has been granted that the subject was of an excessively nervous temperament, of extreme excitability, of an imagination always running into extremes ; dwelling only upon the darkest ideas, affected with fixed fancies, and strangely moved by the least accidents. The majority of children born of such parents were uneven in temper, irritable, coarse, dishonest, sad, difficult to manage, obeying no rule ; scolded, constantly punished, detested by their instructors, in whom they saw their future enemies, the abettors of their persecutions—a form of insanity now so common.

In proportion as we advance in experience, we cannot but deplore the ignorance of men, especially those who are engaged in the instruction of youth. Because they have taken high scholastic rank—because they know Greek and Latin, and have a certain faculty of divining the ordinary intellectual and moral status of their pupils, they consider themselves competent to direct their life-career. Yet there rarely passes a year in which pupils leave the public institutions of whom their masters have neither suspected the talents nor the destined renown. But this is not the question—that with which we chiefly reproach them is, that they ignore completely the physiology of man—that they have not the least knowledge of hereditary influence, and that they believe when they find a pupil idle, captious, or rebellious, that the remedy is perpetually to punish. The first thing ought to be to ascertain if the evil proceed from constitution, from education, or from hereditary causes. In this latter case all chastisement, far from correcting, will only aggravate the evil and hasten the explosion of the disease. I have here for illustration only the embarrassment of choice.

M— —, son of an ill-judging father, who was obstinate, and incapable of directing the education of his children, was from his infancy a witness of and actor in domestic scenes which reacted only too strongly upon an excitable organization. He became sad and pre-occupied, and at twelve years of age he was heard to say he should be glad to die. At school, his melancholy and sulky temper subjected him to frequent punishments; he was expelled, placed elsewhere, again punished, and deprived of his walks. Shut up in his room, he became peevish, rude, singular in manner: at length mental alienation clearly showed itself; and with this disposition it was not surprising that he attempted suicide at sixteen years of age.

A— —, whose mother was deranged, did not lack intelligence, but had the defects of his sad heritage. Similar to the last case in treatment, the result was the same—viz., an access of mania. It was cured, but doubtless a portion of the thorn was left behind.

There is a disease of childhood which exercises evil influence upon the mind of its victims—that is, brain fever. I knew a young man, the son of intelligent and healthy parents, who by their energy and talents had obtained a high position in society. They had six children, five of whom were lively, resolute, and capable of making way in the world. The subject of my observation presented the most marked contrast to his brothers and sisters. Full of good sense, conversing with remarkable justice, he was afflicted with a torpor which nothing could shake. He passed entire days laid upon a sofa, reading everything he could get, without being able to make the least exertion to acquire useful ideas or a suitable education. He listened to advice, declaring at the same time that it was out of his power to follow it. Punished incessantly for idleness by his masters, he never complained, but equally never amended. This young man had had, when twelve months old, a brain fever, which had nearly carried him off. It cannot be doubted that this was the cause of these mental peculiarities; and yet his instructors, who had been informed of this, continually treated him as one of the wilfully bad, making no account of the melancholy physical deterioration. Place this young man in another sphere, that of criminal acts, and justice would only see the accusation and would condemn him! And these facts are frequent.

Let us now resume our examination of antecedents. Physical and moral heritage is not the only influence which has operated unfavourably upon our patients; in ten cases the character had that stamp of singularity and eccentricity which only needed some determining agency to lead to insanity. Continued masturbation and typhoid fever have also, in six cases, seemed to cause mental alienation. In one case it was attributable to the employment of preparations of lead. In another it was the compulsory sight of the execution on the scaffold of a brother, condemned for treason, which brought on furious mania, ending, at twenty-seven years of age, in dementia.

In the female sex there is a function which even in its physiological state ébranle leur moral, et a fait dire de celles qui ont été célèbres qu'elles cessaient d'être hommes. Menstruation is in effect the great regulator of the sex, and when the function is imperfectly performed, especially if there be any hereditary or other predisposing agency, it is frequently the cause of insanity. In the nineteen cases to which we have alluded, twelve times the menstruation, either at the first occurrence, or at the critical period, has exercised a marked influence upon the development of insanity, or of the nervous and hysterical symptoms which have preceded it.

One of these cases appears to us remarkable. A young lady, æt. 15, observed the precursory signs of her first menstruation, and experienced at once the strongest tendency to suicide. Parental affection, judicious care, incessant surveillance, all were lavished upon her; but the idea continued during the flow, became less and less strong as this ceased, but only to reappear with the same intensity at the next period. Esquirol was consulted, and treated her for a year, when the morbid tendency disappeared. Thirty years elapsed without any recurrence; but at the critical period, the same idea recurred with all its pristine force. She was again taken to the private asylum—her reason was perfect upon all other points; but she could not expel the idea of death. She felt herself irresistibly impelled to kill herself—she did not wish it, and made (she said) every effort to resist the tendency, but could not. We observed the case for many months, during which the idea persisted constantly.

In three cases, one of which terminated in idiocy, onanism was the exciting cause. Lastly, the abuse of intoxicating liquors, of *absinthe*, of tobacco, and masturbation, combined to produce insanity in a young man in whose family there was no germ of the affection.

The inquiry into the causes of juvenile insanity, then, shows that it is developed under the same influences as that of adults; only the predisposition receives a fresh impulse from the phenomena of puberty and menstruation.

The *form* of alienation exhibits nothing peculiar. In our cases, seventeen times it was mania, seventeen times monomania. Three other cases were acute delirium—three stupidity

—one was *monomanie orgueilleuse*, and one general feebleness of intellect.

To enter into the symptomatology of these forty-two cases would be to repeat what is found everywhere; there are, however, some particulars which appear worthy of special mention. A merchant feels unable to work, knows that he is ill, and says of his own accord that he has one foot upon the threshold of insanity; he is afflicted at the thought, and would be cured; but he has neither the will nor the power to act.

The *folie de l'orgueil* produces occasionally singular effects. A notary's clerk, well versed in his profession, was attacked by mental alienation. He was formerly fearful, and pusillanimous—he now became bold, hardy, and enterprising—his professional capacity was transformed into an unlimited confidence in his talent and resources. He suggested and invented means of success with an astonishing animation and closeness of reasoning; until we might have been tempted to inquire where was truth and where error, had it not been for a trace of cretinism occurring in the sequel. He ultimately committed an act the audacity of which required his sequestration.

The intermittent form of mania lately described under the name of *folie à double forme*, or "circular insanity," may present so great a calm during the melancholic stage, that we have seen a young man thus affected fulfil with perfect propriety, during four years, his duties in an extensive financial establishment, where he had every day to make the most complex calculations.

Another case became melancholic, and a monstrous *polysarcia* coincided with the period of convalescence; he went out of the house perfectly well, but *double* his former size. Some months afterwards he had returned to his normal condition, and for the last two years his reason has been intact.

One of the most interesting of the cases, and one which we believe at present to be unique in science, by reason of the long period of the incubation of the malady, is that of a man of forty years of age, a distinguished military officer. At thirteen years of age, he was assailed by religious scruples which made him wretched. Six months afterwards these ideas disappeared, and were replaced by that of doing some injury to his parents; this idea occurred on touching some vessel containing verdigris. He thought that the poison adhered to his fingers, and he washed them frequently during the day. This idea persisted for twenty-seven years, often giving him no rest, but never preventing his attending to his duties. At the end of this time it became more intense; he felt that he was losing command over himself; and at length, accompanied by one of his relatives, he came to relate his sufferings to me—sufferings which neither his present companion nor any of his friends had ever suspected. What surprised me the most was, that after three months of care, he was restored to calmness. He was able to accomplish a mission of importance, and I saw him many years afterwards in the most perfect soundness of mind.

We have likewise attended a young lady who for a long time, at the periods of menstruation, was pursued by the idea of doing some evil. She could not see a knife or a fork at table without this idea being intensified. It then seemed to her that her hands were red with blood, and she kept perpetually washing them, whilst none of the family could conjecture the motives for this exaggerated cleanliness.

Religious scruples are very common amongst young girls, and their alarmed consciences easily lead them to believe themselves eternally lost. In these cases preaching and imprudent forms of tuition may have the most disastrous consequences.

Hysterical symptoms of all kinds are very frequent as the forerunners of mental alienation; in concert with menstruation, these symptoms assume the most varied forms, and are complicated with epileptiform and cataleptic attacks; and we do not hesitate to assert that we have verified in some instances certain of the phenomena of animal magnetism. This subject, a very delicate one, is about to be entered upon by the Medico-Psychological Society; and we believe that it is quite time that capable and conscientious men should examine into the question, as to what really scientific elements can be deduced from this part of our knowledge, as yet very obscure. When men of such eminence and learning as MM. Ferrus, Cérise, Peisse, Des Etanges, and others, shall have given the result of their experience, we shall then have a good criterion as to the merits of magnetism.

Amongst the nervous symptoms of the hysteric character, we must not forget a special form of convulsive cough, which we have observed in four cases of insanity. It may persist for months, even for years after recovery; then it becomes intermittent, and disappears as it came.

When the delirious idea flickers about the mind, we generally advise the patient to repel it, and make no concession to it; this rule, however, is subject to exception. In the case of one young female patient, each time that she attempted to repel the morbid idea, of which she recognised the falsity, or to dissimulate it, in order to avoid remonstrances, she was subject to extreme agitation and spasm, to a sort of convulsive action; she rubbed her hands and thighs with extreme rapidity, and wore out her clothes without being aware of it.

Sometimes we observe in young girls very odd habits. One young lady perpetually pulled her front hair over her face; it was cut off once for the purpose of breaking her of the habit; but as soon as it grew again she resumed it. She would also walk five steps forward and five backwards for hours together.

Apathy and indifference are sometimes carried to the extreme. Some of the patients would remain for entire days in one position, as if stupified, and would not occupy themselves in any manner, unless absolutely compelled to it. They lamented this state, but affirmed that they could not move. Others, again, would exhibit a vivacity by no means natural to them, would express themselves in terms more polished than usual, would speak on subjects not generally familiar to them—it was a veritable metamorphosis. These changes of character have been brought about frequently by typhoid fever, the influence of which in these respects has not been sufficiently indicated.

To form a prognostic sufficiently accurate upon this form of mental alienation, it would be necessary to enter upon the medical biography of each of these cases; to follow the course of the antecedents, the incubation, the relapses, the progress, the termination, and the consequences of the affection. This we shall do in some detail, leaving out such of the antecedents as have been before treated of.

In nine individuals, the disorders of mind of which the manifestation dated from puberty, had either been restrained, concealed, or showed themselves with such characters that isolation had not been necessary, nor medical advice taken. The period of incubation varied in these cases from four to twenty-seven years. This last fact is full of interest, inasmuch as the patient, during this long period was able to conceal his sufferings from the penetrating eyes of his relatives and associates. Seven times the relapse occurred at intervals of from nine to forty-one years. In two observations, where there had been an interval of thirty years in each case, the critical period was the determining cause of the relapse. In three others, it was accouchement. One of these ladies, after an interval of eleven years, has fallen ill again.

If we analyse now the progress, the consequences, and the terminations of the derangements, we find amongst the twenty-three men the following results:—

| | |
|---|---|
| Dead in a state of dementia . . . . . | 2 |
| Cured—Died of bronchitis . . . . . . | 1 |
| Left melancholic—lost sight of . . . . | 3 |
| Commencing dementia . . . . . . . | 1 |
| Fallen into a state of idiocy . . . . . . | 1 |
| Commencing feebleness of intellect . . . . | 1 |
| Remained melancholic . . . . . . . | 1 |
| Cured . . . . . . . . . . . | 13 |
| | 23 |

This last result (viz., the thirteen cured) requires further attentive analysis.

| | |
|---|---|
| Of these, 3 were lost sight of, and 1, an artist of great merit, died of fever many years after his cure . . . . | 4 |
| Six others had either relapses, or remained quarrelsome, unmanageable, inconstant, uncertain, and changeable—some of the 6 were drunkards and masturbators, and had, as consequences of these vices, epileptiform attacks . . . . . | 6 |
| There remain satisfactorily cured . . . . . . . . | 3 |
| | 13 |

Let us now pursue the same analysis amongst the females, and see what will be the result of the examination as regards prognosis.

The nineteen females, of whom five were married, may be thus divided :—

| | |
|---|---|
| Four left uncured, with forms of insanity of an intractable nature, or presenting phenomena of a nervous or hysterical kind. They have been lost sight of . . . . . | 4 |
| Two relapsed—one after 11 years, the other after 30, presenting the same symptoms as at first . . . . . . . | 2 |
| Six strove for many years against their delirious ideas without success; finding, on the contrary, that they ever increased in intensity. Two of these were like automatons, so apathetic were they and indifferent to everything . . . | 6 |
| One fell into dementia . . . . . . . . . . | 1 |
| Six were cured—three after having had two, three, and four relapses. The others were only treated for the first attack, and have been lost sight of. All these, except one, were of a difficult, uncertain, fantastic character, excitable, and weak of judgment . . . . . . . . . | 6 |
| | 19 |

If we have entered thus minutely into this examination, it is because it was of importance to know what data we might calculate upon in forming a prognosis of the insanity of young persons, especially those born of parents so affected, or in whose families or history there existed the elements of insanity. Far from us be the wish to extend this melancholy influence beyond its due limits, as has been done of late years by means of the theory of "pathological transformations." We have sought for mental alienation where it was, and have carefully avoided doubtful cases. What has our examination revealed to us?

Eighteen times out of forty-two, the children inherited the mental malady from their parents, or from their eccentric and *bizarre* habits. In the great majority of cases, either under the hereditary influence, or under that of puberty or menstruation, we have recognised the principle of the elements of mental alienation. In interrogating the parents upon the characters of their children, they have almost always answered that they were sad or gay without motive; they could not fix them to work; they had not capacity; or perhaps occasionally they had brilliant talents, but could not submit to any rule. Some were apathetic, without emulation; others of a frivolity which nothing could control. Many had convulsive affections. A very long period of incubation presaged a grave malady. The eighteen cures obtained had been often preceded by relapses—left often great changes in the character, especially an inaptitude to assume and keep any defined position in society, and presented but uncertain chances of durability of cure. The consequence deducible from this summary is, that if the cure be permanent in some cases (which we are far from denying), yet mental alienation in the young is a malady of very grave import, whether by reason of the antecedents, or of the incomplete development of the organism.

We have not spoken of the treatment, because our therapeutic agents are those of all enlightened practitioners; but especially because in these cases, it is chiefly *preventive* measures to which recourse should be had. A distinguished physician who, in his treatise upon the "Degenerations of the Human Race," has had the merit of opening a new method in our science, eminently social and anthropological, has insisted strongly upon the form of degeneration produced by mental alienation.

Eighteen years ago, in a memoir read before the Academy of Sciences, we called the attention of the learned world to the progress of insanity. This opinion, controverted for some time, is now admitted by many authorities.* All civilized States, where charity is at the height of progress, have seen their magnificent establishments scarcely opened, but crowded at once with the insane; yet without are thousands similarly affected—not dangerous—idiots and cretins, who have an equal right to admission, as they are equally the victims of the ignorance and prejudices of society. Hereditary influence! there is in reality the knot of the question—that upon which we should direct all our efforts—that which has impelled us in these researches. When we have grouped the facts related in scattered works—when we have added our statistics to the valuable labours of Dr. Lucas upon "Natural Inheritance"—when we have proved to all that insanity is transmitted fatally by the seminal germ in very large proportion—when we have exactly realized the strong bond of

* Once for all, we do not attack civilization and progress; we only mark out the events which embarrass their march.

union between this and other nervous affections, and the generative influence of these latter in producing the former, we may then, with hope of success, occupy ourselves with the social measures of hygiene necessary to be adopted to arrest this degeneration. Even now, it is ascertained that drunkenness engenders mental alienation, and creates thousands of idiots, imbeciles, and feeble-minded persons. The facts cited by Dr. Magnus Huss and M. Morel* leave no doubt on this point. M. Ferrus, in his book on Prisoners, has likewise shown that the prisons contain a considerable number of these degenerate beings, the feeble nature of whose faculties place them in the power of clever scoundrels, whose instruments they are. No session ever passes where we do not observe criminals with lowering brow, fixed, dogged look, and imbecile physiognomy, with dangerous instincts and habits, who listen unmoved to their sentence, as though it in no wise concerned them. It is not many months since one of these wretched creatures was condemned to the galleys for having murdered a child, in order to *become invisible* (!) that he might rob with impunity! When the scientific facts, of which we are now the depositaries, shall have passed from our books to the public, and make part of the public education, so far behind-hand in the practice of life, then physicians will be called in to examine such criminals, and their reports will show, in many such cases, the hereditary result of drunkenness, of imbecility, and of mental alienation.

Meantime our mission is incessantly to indicate the *preventive cure* of insanity; to oppose all our forces to the causes of degeneration; to prevent the affected from perishing, and the sound from becoming affected by contact with the unsound. Observation also shows that we may combat the degeneration of the race by crossing the breed of races. The facts illustrative of this position are as yet chiefly derived from the domestic animals. Without going out of France, and confining ourselves to two recent experiments, we may mention the race of "charmoise" sheep, and the pigs of Bologne. The former are produced by a somewhat elaborate double crossing of different breeds, by which a race is obtained double in value to that of the parents. The pigs of Bologne are derived from an extremely degraded local breed, which are crossed with Yorkshire and Leicester pigs. The *mules* thus obtained breed together, and so a very fine breed, furnishing an important article of commerce, is obtained. With regard to the objections to crossing of races, it is sufficient to say that the want of success has been chiefly due to the neglect of the most ordinary

* A summary of these will be found in our last number, "On the Degeneracy of the Race."

physiological laws; as, for instance, in the attempt to mix our race of horses with that of the English.

However cautious we ought to be in any comparisons between men and animals, we believe that these facts should be taken into consideration. There are, moreover, experiments ready made in the human race, which throw much light on the subject.

Wherever precise observations have been made, the mixed races are found superior to the coloured—almost equal, sometimes superior in certain respects, to the white races themselves. In the Philippine Islands, the mixed race forms a numerous, active, brave, industrious class of people, who have already obtained many and just concessions. It is scarcely necessary to recal what were those men of colour at St. Domingo, who so cruelly expiated their alliance with the blacks.

In Brazil, thanks to its moral and intellectual force, the crossed race has in great measure overcome the prejudice of blood, and it is especially remarkable for an aptitude for cultivation of the arts far superior to that of the pure white race. In this same empire, we find an entire province entirely peopled by a breed or race, a cross between the Europeans and the indigenous inhabitants. What is the result of this marriage? Their peculiar stamp, their chivalrous character, their bravery, and their perseverance, have been recounted by M. Quatrefages in his "Histoire naturelle de l'Homme."*

Marriage is, then, the great preventive of insanity. Such a subject can only be slightly hinted at in a memoir like the present; we shall treat of it in speaking of the means necessary to be opposed to the development of alienation in general. Our observations upon the insanity of young people furnish one page of the history of mental maladies; we trust our fellow-labourers will give them a favourable reception.

---

